# Genome analysis of secondary metabolite‑biosynthetic gene clusters of *Photorhabdus akhurstii* subsp. *akhurstii* and its antibacterial activity against antibiotic-resistant bacteria

**DOI:** 10.1371/journal.pone.0274956

**Published:** 2022-09-21

**Authors:** Paramaporn Muangpat, Wipanee Meesil, Jatuporn Ngoenkam, Yothin Teethaisong, Rapee Thummeepak, Sutthirat Sitthisak, Sarunporn Tandhavanant, Narisara Chantratita, Helge B. Bode, Apichat Vitta, Aunchalee Thanwisai

**Affiliations:** 1 Faculty of Medical Science, Department of Microbiology and Parasitology, Naresuan University, Phitsanulok, Thailand; 2 Faculty of Allied Health Sciences, Department of Biomedical Sciences, Burapha University, Chonburi, Thailand; 3 Research Unit for Sensor Inovation (RUSI), Burapha University, Chon Buri, Thailand; 4 Faculty of Tropical Medicine, Department of Microbiology and Immunology, Mahidol University, Bangkok, Thailand; 5 Molekulare Biotechnologie, Goethe Universität Frankfurt, Frankfurt am Main, Germany; 6 Department of Natural Products in Organismic Interactions, Max Planck Institute for Terrestrial Microbiology, Marburg, Germany; 7 Faculty of Sciences, Center of Excellence for Biodiversity, Naresuan University, Phitsanulok, Thailand; 8 Faculty of Medical Science, Centre of Excellence in Medical Biotechnology (CEMB), Naresuan University, Phitsanulok, Thailand; Indian Agricultural Research Institute, INDIA

## Abstract

*Xenorhabdus* and *Photorhabdus* can produce a variety of secondary metabolites with broad spectrum bioactivity against microorganisms. We investigated the antibacterial activity of *Xenorhabdus* and *Photorhabdus* against 15 antibiotic-resistant bacteria strains. *Photorhabdus* extracts had strong inhibitory the growth of Methicillin-resistant *Staphylococcus aureus* (MRSA) by disk diffusion. The *P*. *akhurstii s* subsp. *akhurstii* (bNN168.5_TH) extract showed lower minimum inhibitory concentrations (MIC) and minimal bactericidal concentrations (MBC). The interaction between either *P*. *akhurstii* subsp. *akhurstii* (bNN141.3_TH) or *P*. *akhurstii* subsp. *akhurstii* (bNN168.5_TH) or *P*. *hainanensis* (bNN163.3_TH) extract in combination with oxacillin determined by checkerboard assay exhibited partially synergistic interaction with fractional inhibitory concentration index (FICI) of 0.53. Time-killing assay for *P*. *akhurstii* subsp. *akhurstii* (bNN168.5_TH) extract against *S*. *aureus* strain PB36 significantly decreased cell viability from 10^5^ CFU/ml to 10^3^ CFU/ml within 30 min (P < 0.001, t-test). Transmission electron microscopic investigation elucidated that the bNN168.5_TH extract caused treated *S*. *aureus* strain PB36 (MRSA) cell membrane damage. The biosynthetic gene clusters of the bNN168.5_TH contained non-ribosomal peptide synthetase cluster (NRPS), hybrid NRPS-type l polyketide synthase (PKS) and siderophore, which identified potentially interesting bioactive products: xenematide, luminmide, xenortide A-D, luminmycin A, putrebactin/avaroferrin and rhizomide A-C. This study demonstrates that bNN168.5_TH showed antibacterial activity by disrupting bacterial cytoplasmic membrane and the draft genome provided insights into the classes of bioactive products. This also provides a potential approach in developing a novel antibacterial agent.

## Introduction

Methicillin-resistant *Staphylococcus aureus* (MRSA) is a predominant aetiology of hospital and community-acquired infections, with higher mortality compared to susceptible strains. MRSA can infect in different parts of our body, including the bloodstream, lower respiratory tract, skin and soft tissues, ventilator-associated pneumonia and central venous catheter-associated bacteremia [1]. In addition to appropriate antimicrobial therapy, infectious disease consultation reduces the mortality from MRSA bacteremia is associated with reduced mortality [2]. Due to the increased incidence of antimicrobial resistance and limited prospect of discovery of novel antimicrobial compounds, the effective use of antimicrobials in the future has become uncertain [3]. Finding of natural compounds from new antibacterial agents is important for alternative treatment of antibiotic-resistant bacteria.

*Xenorhabdus* and *Photorhabdus* are Gram-negative bacilli belonging to the family Morganellaceae [4]. They are associated with the infective juveniles of entomopathogenic nematodes in the genera *Steinernema* and *Heterorhabditis*, respectively. Currently, 27 species of *Xenorhabdus* and 21 species of *Photorhabdus* were documented worldwide [4–10]. These bacteria are known to produce numerous secondary metabolites that show bioactivity against bacteria, fungi, insects, nematodes, and protozoa [11–13]. *Xenorhabdus* and *Photorhabdus* can produce several antimicrobial compounds, including xenocoumacins [14] and indole derivatives [15], xenortides [16], bicornutin [17], isopropylstilbenes [18], Ethylstilbene [18], anthraquinones [18], szentiamide [19], xenoamicins [20] and Fabclavines [21] and several antimicrobial peptides (AMPs), such as PAX peptide [22], xenobactin [23], and, rhabdopeptides [24], cabanillasin [25], and taxlllaids [26]. Therefore, these bacteria are interest source for novel natural products.

A previous study revealed that, the cell filtrates of *Xenorhabdus* and *Photorhabdus* had an inhibitory effect on the growth of various plant pathogenic fungi, including *Botrytis cinerea*, *Ceratocystis ulmi*, *Ceratocystis dryocoetidis*, *Mucor piriformis*, *Pythium coloratum*, *Pythium ultimum*, and *Trichoderma pseudokingii* [27]. Subsequently, Fang et al. [28] reported the cell-free filtrate of *Xenorhabdus bovienii* YL002 exhibited high antifungal effect on *Phytophthora capsica*, *Botrytis cinerea*, *Dothiorella gregaria*, *Bipolaria maydis*, *Sclerotinia sclerotiorum*, *Bipolaris sorokinian* and *Rhizoctonia cerealis*. In addition, *Xenorhabdus* and *Photorhabdus* extracts were observed for their antimicrobial activity against *P*. *cactorum*, *Fusicladosporium effusum*, *Monilinia fructicola* [29] and *S*. *aureus* (MRSA) [30–32]. Moreover, the antimicrobial activity of secondary metabolite compound can inhibit the growth of *F*. *effusum* [33], *P*. *capsici*, *Rhizoctonia solani*, *Corynespora cassiicola* [34], *S*. *aureus* (MRSA) [13], *Escherichia coli*, *Klebsiella pneumoniae* and *Enterobacter cloacae* [35].

The *P*. *laumondii* subsp. *laumondii* strain TT01 was the first to be completely sequenced [34]. Several gene-encoding enzymes involved in secondary metabolite biosynthesis were identified, including antibiotic synthesising genes and encoding a large number of adhesins, insecticidal proteins, hemolysins, protease and lipase [18,36]. Genomic and metabolic characteristics of 25 *Xenorhabdus* and 5 *Photorhabdus* were identified and elucidate an additional class of natural product [37]. A large number of resources for the production of specialised metabolites derived from non-ribosomal peptide synthetase (NRPS) or polyketide synthase (PKS) were identified [37]. This study aims to study the antibacterial activities of *Xenorhabdus* and *Photorhabdus* against 15 strains of antibiotic-resistant bacteria. Further, the study on the morphology of *S*. *aureus* (MRSA) and cell line toxicity after treating the bacterial extract were studied. In addition, the whole genome of *P*. *akhurstiis* subsp. *akhurstii* was analyzed to identify the secondary metabolite gene cluster.

## Materials and methods

### Preparation of antibiotic-resistant bacteria

The protocol on bacterial culture, biotechnology and biological safety was approved by the Naresuan University Institutional Biosafety Committee (NUIBC MI62-06-25). In this study, fifteen strains of antibiotic-resistant bacteria were selected for determination of antibacterial activity, including *Acinetobacter baumannii* (four clinical strains), *Escherichia coli* (three clinical strains), *E*. *coli* ATCC35218, *Klebsiella pneumoniae* (two clinical strains), *K*. *pneumoniae* ATCC700603, *Enterococcus faecalis* ATCC51299, *Staphylococcus aureus* (two clinical strains) and *S*. *aureus* ATCC20475 (Table 1). The individual bacterial strain was streaked on the Mueller-Hinton agar (MHA) and incubated at 37°C for 24 h. A single colony was dissolved in 0.85% normal saline solution, and the concentration was adjusted to 0.5 McFarland standards [38].

**Table 1:** Disc diffusion of ethyl acetate extracts of *Xenorhabdus /Photorhabdus* against antibiotic-resistant bacteria.

Bacteria list (Code)	Inhibit the growth of antibiotic-resistant bacteria														
	*A*. *baumannii* (XDR)^a^ strain AB320		*A*. *baumannii* (MDR) ^b^ strain AB322			*S*. *aureus* (MRSA)^c^ strain PB36									
		*A*. *baumannii* (MDR) ^b^ strain AB321		*A*. *baumannii* (XDR) ^a^ strain AB324	*S*. *aureus* ATCC20475		*S*. *aureus* (MRSA)^c^ strain PB57	*E*. *coli* ATCC35218	*E*. *coli* (ESBL+MDR) ^d^^,^^b^ strain PB1	*E*. *coli* (ESBL+CRE) ^d^^,^^e^ strain PB231	*E*. *coli* (MDR)^b^ strain PB30	*E*. *faecalis* ATCC51299	*K*. *pneumoniae* ATCC700603	*K*. *pneumoniae* (ESBL+MDR) ^d^^,^^b^ strain PB5	*K*. *pneumoniae* (ESBL+CRE) ^d^^,^^e^ strain PB21
*X*. *stockiae* (bNN94.5_TH)	-	-	-	-	-	-	-	-	-	-	-	-	-	-	-
*P*. *akhurstii* subsp. *akhurstii* (bNN141.3_TH)	-	-	-	-	++	++	++	-	-	-	-	++	-	-	-
*P*. *hainanensis* (bNN163.3_TH)	+	+	+	-	++	++	++	-	-	-	-	++	-	-	-
*X*. *vietnamensis* (bNN167.3_TH)	-	-	-	-	+	+	+	-	-	-	-	+	-	-	-
*P*. *akhurstii* subsp. *akhurstii* (bNN168.5_TH)	+	++	+	-	++	++	++	-	-	-	-	++	-	-	-
*P*. *hainanensis* (bNN169.4_TH)	+	+	+	-	+	++	++	-	-	-	-	++	-	-	-
*X*. *stockiae* (bNN175.2_TH)	+	+	-	-	-	+	+	-	-	-	-	-	-	-	-

- No activity (6 mm), + weak inhibition (7–10 mm.), ++ moderate/average inhibition (11–15 mm.), strong inhibition (16–20 mm.)

^a^Extreme drug resistance

^b^ multidrug resistance

^c^ methicillin resistance *Staphylococcus aureus*, and

^d^ extended-spectrum beta-lactamase

^e^Carbapenem-resistant Enterobacteriaceae.

### Screening of *Xenorhabdus* and *Photorhabdus* isolates

*Xenorhabdus* (11 isolates) and *Photorhabdus* (12 isolates) were used in this study. *Xenorhabdus* were isolated from *Steinernema* and *Photorhabdus* isolated from *Heterorhabditis*. These entomopathogenic nematodes were collected from the soil samples at the Nam Nao National Park of the Phetchabun province, Thailand [39]. These bacteria were cultured on nutrient bromothymol blue triphenyl tetrazolium chloride agar (NBTA) for four days at room temperature and transferred into Luria-Bertani (LB) broth for shaking at room temperature for 48 h. The whole-cell suspension of these bacteria was used for the screening of antibacterial activity. Twenty microliters of the whole-cell suspension were dropped on the mueller hinton agar (MHA) plated with antibacterial-resistant bacteria. The plates were then incubated at 37°C for 24 h. An inhibition zone diameter from the edge of the growth colony of *Xenorhabdus* and *Photorhabdus* was read as positive. The *Xenorhabdus* and *Photorhabdus* isolates that showed potential inhibition the growth of antibiotic-resistant bacteria was further selected for extraction in the disk diffusion test.

### Bacterial extracts

Three isolates of *Xenorhabdus* and four isolates of *Photorhabdus* show potent antibacterial activity in the screening technique used to extract bacterial extracts. A single colony of *Xenorhabdus* and *Photorhabdus* on NBTA was inoculated in a 1,000 ml flask containing 500 ml of LB broth. The flask was incubated at room temperature with shaking at 180 rpm for 72 h. Subsequently, 1,000 ml of ethyl acetate was added to the culture and mixed well. The flask was then allowed to stand at room temperature for 24 h. Extraction with the respective solvent was performed three times by a rotary vacuum evaporator (Buchi, Flawil, Switzerland). The extract was dried, and condensed extracts were weighted and kept at -20°C until used.

### Disk diffusion method

Ten microliters of 7 isolates of bacterial extract (500 mg/ml) were added to a paper disc (6-mm diameter of Whatman no. 3) and air-dried. A sterile cotton swab was dipped in the antibiotic-resistant bacterial suspension on 0.85% normal saline solution and was streaked over the entire surface of the MHA medium, ensuring an even distribution of the inoculum. The antimicrobial discs (vancomycin, tigecycline, ampicillin, ceftazidime, and ceftazidime/clavulanic acid (Oxoid, England)) and DMSO were used as positive and negative controls, respectively. Then, they were incubated at 37°C for 24 h. After incubation, inhibition zone diameter was measured in millimetres using a ruler. Two independent experiments of disk diffusion assay were performed.

### Minimum inhibitory concentrations (MIC) and minimal bactericidal concentrations (MBC)

Minimum inhibitory concentrations (MIC) were performed on a 96-well microtiter plate by broth dilution method. The concentration of the bacterial extract was diluted in two-fold dilution with a Cation-Mueller-Hinton broth (CaMHB), ranging from 250 mg/ml to 0.98 mg/ml. Afterwards, antibiotic-resistant bacterial culture was added to each well to get a final concentration of 10^6^ CFU/ml. Positive controls were the mixture of bacterial suspension and CaMHB and bacteria suspension and DMSO, and negative control was CaMHB. The microtiter plate was incubated at 37°C for 24h. The MIC value was defined as the destruction or absence of bacterial growth at the minimum concentrations of different extracts. After the determination of MIC, ten μl from each well of 96-well microtiter plates were dropped into the MHA to investigate the effective concentration of bacterial extract. The plate was then incubated at 37°C for 24 h. The lowest concentration of each extract without the growth of bacteria was considered as MBC. MIC and MIBC assay were tested in two replicates.

### Checkerboard determination

Antimicrobial combinations were performed following Teethaisong et al. [40]. Two independent experiments were performed. The cultured and antibacterial agents were prepared and performed similarly with MIC determination. A total of 50 μl of CaMHB was distributed into each well of 96-well microtiter plate. The antibiotics (vancomycin and oxacillin) of the combination were serially diluted along the ordinate, while the bacterial extracts were diluted along the abscissa. Each well was inoculated with 100 μl of an *S*. *aureus* strain PB36 suspension (0.5 MacFarland standard) inoculum, and the plates were incubated at 37°C for 24 h. The resulting checkerboard contained the lowest concentration of two antibiotics. The fractional inhibitory concentration index (FICI) was calculated as follows: FIC index = FIC bacterial extract + FIC antibiotic, where FIC bacterial extract is the MIC of bacterial extract in the combination/MIC of bacterial extract alone and FIC antibiotic is the MIC of antibiotic in the combination/MIC of antibiotics alone. The results were interpreted as follows: FICI ≤ 0.5, synergistic; 0.5 < FICI < 1, partially synergistic; FICI = 1, additive; >1 FICI ≤ 4, indifferent; and FICI > 4, antagonistic [41].

### Time-killing assay

Bacterial extract at a MIC concentration was mixed with the *S*. *aureus* strain PB36 cultured in the CaMHB and then adjusted to a final inoculum of 10^5^ CFU/ml. The mixture was diluted and counted using a drop plate in time 0, 0.5, 1, 2, 3, 4, 5, 6 h and then 24 h on MHA plates. The plate was then incubated at 37°C for 24 h. After incubation, the lowest detectable limit for counting is 10^3^ CFU/ml. Significant differences of the *S*. *aureus* strain PB36 (MRSA) treated with the bacterial extracts at different times were analyzed by t-test (Stata version 13). Time-killing assay was tested in two replicates. The *p* < 0.001 was considered as the statistically significant difference.

### Transmission electron microscopy (TEM)

The samples were prepared following Teethaisong et al. [40]. First, the *S*. *aureus* strain PB36 was grown in antibiotic-free (control) and bacterial extract alone at ¾ MIC to get a final concentration of 5 x 10^5^ CFU/mL for 4 h with shaking 150 rpm at 37°C. The culture was centrifuged at 6,000 rpm for 15 min at 4°C and fixed in 2.5% glutaraldehyde for 12 h. The sample was washed twice with 0.1 M phosphate buffer (pH7.2), and post-fixation was carried out with 1% osmium tetroxide for 2 h at room temperature. After washing in the buffer, the samples were gently dehydrated with acetone solutions (20%, 40%, 60%, 80% and 100%, respectively) for 15 min. Afterwards, infiltration and embedding were performed using Spurr’s resin (electron microscope sciences; EMS); the block resin was thin-sectioned by Leica EM UC7 (Heerbrugg, Switzerland) and mounted on copper grids. Finally, the ultrathin sections were counterstained with 2% (w/v) uranyl acetate for 3 min and then 0.25% (w/v) lead citrate for 2 min. Following staining, the specimens were visualised, and images were captured with a Hitachi HT7700 Transmission electron microscope (Tokyo, Japan), operating at 80 kV.

### The 3-(4,5-dimethylthiazol-2-yl)-2,5-diphenyl-2H-tetrazolium bromide (MTT) assay

MTT was prepared following Impheng et al. [42] with some modification. The human hepatocellular carcinoma HepG2 cell line was cultured in Dulbecco’s modified Eagle’s medium (DMEM), supplemented with 10% fetal bovine serum (FBS), 100 U/ml penicillin and 100 μg/ml streptomycin at 37°C in 5% CO_2_ and 95% humidity. The HepG2 cells were seeded into a 96-well plate at a density of 1 × 10^4^ cells/well. Cells were allowed to adhere overnight and were then treated for 24 h with various concentrations of bacterial extract diluted with complete DMEM to give a final concentration of 0–7.81 mg/ml. The control samples were cultured in a complete DMEM medium containing 0.2% DMSO. After incubation, 20 μL of MTT solution (5 mg/mL in PBS) (Tokyo Chemical Industry Co., Ltd., Japan) was added to each well and incubated further for 2 h at 37°C. The cultured medium was removed, formazan crystals formed by viable cells were dissolved in 100 μl of DMSO, and absorbance was measured at 590 nm using a microplate spectrophotometer. The percentage of cell viability was calculated in comparison to the control group, which was arbitrarily assigned 100% viability. The IC50 of the extract from the bacterial culture medium was defined as the concentration of the extract that caused a 50% reduction in cell viability compared with the control using Graph Pad Prism version 5 [42]. MTT assay was tested in three independent experiments with triplicate wells for each condition. One-way analysis of variance (ANOVA) with Tukey’s comparison test was used to assess the statistically significant differences among the experimental group.

### Genome sequencing and annotation

#### DNA extraction and preparation

*P*. *akhurstii* subsp. *akhurstii* (bNN168.5_TH) was grown in 5 ml LB for 24 h. The genomic DNA was extracted using a QIAamp DNA Mini Kit (Qiagen, Germany). Extracted DNA was roughly quantified using a nanoDrop spectrophotometer (Thermo Scientific‎). After performing quality control (QC), the qualified sample proceeded to library construction. Sequencing libraries were prepared using the Nextera XT DNA Library Preparation Kit before sequencing on HiSeq 4000-100PE instrument. This service was delivered by Macrogen (Seoul, Korea). After sequence data generation, paired-end raw reads were processed using FastQC v. 0.72 to assess data quality. The sequencing reads were then trimmed using Sickle v. 1.33.2 to remove sequencing adapters.

#### De novo genome assembly and annotation

Genome assembly was performed using the SPAdes v. 3.12.0 software. Reads were initially normalised with k-mer 21, 33, 55, 77, 99 and 127. Finally, the assembly was performed using the recommended parameters for such Illumina data. The SPAdes software produces a contigs file, whereas removal of poor-quality reads < 500 bp was done using the filter sequences by length v. 1.2. Post-assembly correction of sequences length up to 500 bp was generated using the Pilon v. 1.22 with default settings. The RAST tool kit (RASTtk) was used for genome annotation and gene prediction.

#### Comparative genomic analysis

The closely related species obtained from the NCBI datasets were used as reference strains. To determine which species is the closest to *P*. *akhurstii* subsp. *akhurstii* (bNN168.5_TH), a genome-based phylogenetic tree of these species with *P*. *akhurstii* subsp. *akhurstii* (bNN168.5_TH) was constructed using the REALPHY online tool. Moreover, ANI calculations among *P*. *akhurstii* subsp. *akhurstii* (bNN168.5_TH) and other *Photorhabdus* strains were performed using FastANI v. 1.3.

#### Secondary metabolite cluster identification

AntiSMASH v. 5.1.2 was used for a rough secondary metabolite biosynthesis gene cluster (BGCs) with the optional ClusterFinder algorithm using the annotated genomes as input.

## Results

### Effect of *Xenorhabdus/Photorhabdus* on antibiotic-resistant bacteria

**Fig 1:**
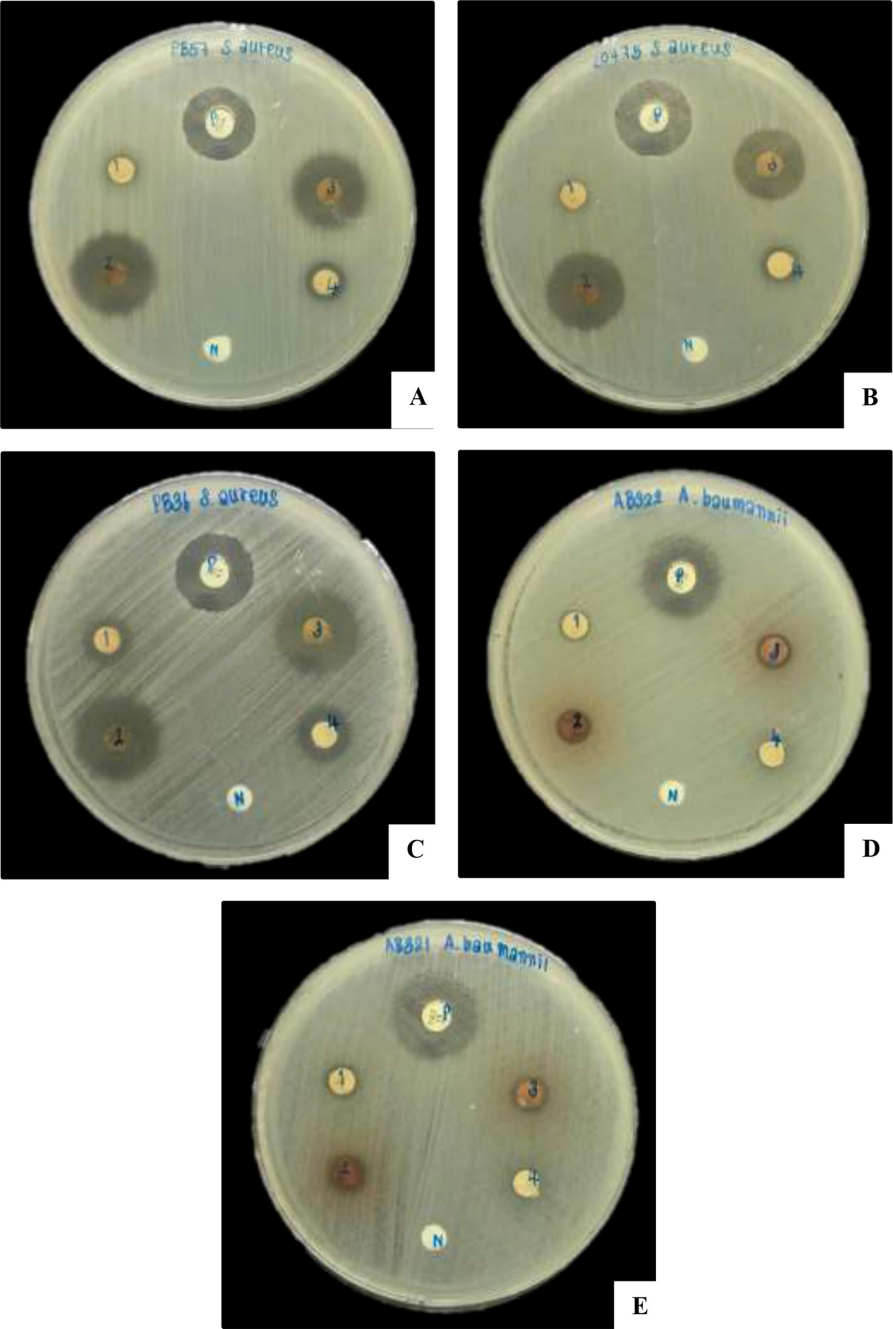
Disk diffusion of ethyl acetate extracts of *Xenorhabdus /Photorhabdus* against five antibiotic-resistant bacteria. The inhibition zone of *S*. *aureus* strain PB57 (MRSA; A), *S*. *aureus* ATCC20475 (B), *S*. *aureus* strain PB36 (MRSA; C), *A*. *baumannii* strainAB322 (MDR; D) and *A*. *baumannii* strain AB322 (MDR; E) after exposure to bacterial extracts from *X*. *stockiae* bNN94.5_TH (1), *P*. *akhurstii* subsp. *akhurstii* bNN141.3_TH (2), *P*. *hainanensis* bNN163.3_TH (3), *X*. *vietnamensis* bNN167.3_TH (4), antibiotic discs (P) and negative control (N).

Bacterial extraction from 3 isolates of *Xenorhabdus* and 4 isolates of *Photorhabdus* (Table 1 and Fig 1), including *X*. *stockiae* (bNN94.5_TH, and bNN175.2_TH), *X*. *vietnamensis* (bNN167.3_TH), *P*. *akhurstii* subsp. *akhurstii* (bNN141.3_TH, and bNN168.5_TH) and *P*. *hainanensis* (bNN163.3_TH, and bNN169.4_TH) were tested against 15 strains of antibiotic-resistant bacteria by disk diffusion method. The result shows that four bacterial extracts, including *P*. *akhurstii* subsp. *akhurstii* (bNN141.3_TH, and bNN168.5_TH) and *P*. *hainanensis* (bNN163.3_TH, and bNN169.4_TH), could inhibit the growth of *A*. *baumannii* strain AB321 (MDR), *A*. *baumannii* strain AB322 (MDR), *S*. *aureus* ATCC20475, *S*. *aureus* strain PB36 (MRSA), *S*. *aureus* strain PB57 (MRSA) and *E*. *faecalis* ATCC51299 better than bacterial extracts of *Xenorhabdu*s spp. The similar results were obtained from 2 independent experiments.

The MIC and MBC were tested for *Photorhabdus* against five strains of antibiotic-resistant bacteria including *S*. *aureus* strain PB36 (MRSA), *S*. *aureus* strain PB57 (MRSA), *A*. *baumannii* strain AB321 (MDR), *A*. *baumannii* strain AB322 (MDR) and *E*. *faecalis* ATCC51299. Two isolates of *P*. *hainanensis* (bNN163.3_TH and bNN169.4_TH) and two isolates of *P*. *akhurstii* subsp. *akhurstii* (bNN141.3_TH and bNN168.5_TH) extracts against five strains of antibiotic-resistant bacteria had the MIC and MBC ranging from 0.98 to 31.25 mg/mL (Table 2). All 4 *Photorhabdus* extracts against *S*. *aureus* strain PB36 (MRSA) and *S*. *aureus* strain PB57 (MRSA) showed lowest MIC and MBC as 0.98 mg/mL. In addition, the *P*. *akhurstii* subsp. *akhurstii* (bNN168.5_TH) extract shows the lowest MIC and MBC against all antibiotic-resistant bacteria. The same result of two independent experiments of MIC and MBC were obtained.

**Table 2:** Antibacterial activity of the *Photorhabdus* extracts against antibiotic-resistant bacteria as assessed by minimum inhibitory concentrations and minimal bactericidal concentrations (mg/ml).

Bacteria list (code)	Concentration of inhibition (mg/ml)									
	*S*. *aureus* (MRSA) strain PB36		*S*. *aureus* (MRSA) strain PB57		*A*. *baumannii* (XDR) strain AB321		*A*. *baumannii* (MDR) strain AB322		*E*. *faecalis* ATCC51299	
	MIC	MBC	MIC	MBC	MIC	MBC	MIC	MBC	MIC	MBC
*P*. *akhurstii* subsp. *akhurstii* (bNN141.3_TH)	0.98	0.98	0.98	0.98	15.62	31.25	15.62	31.25	31.25	31.25
*P*. *hainanensis* (bNN163.3_TH)	0.98	0.98	0.98	0.98	ND	ND	ND	ND	3.90	7.81
*P*. *akhurstii* subsp. *akhurstii* (bNN168.5_TH)	0.98	0.98	0.98	0.98	0.98	0.98	1.95	1.95	3.90	7.81
*P*. *hainanensis* (bNN169.4_TH)	0.98	0.98	0.98	0.98	3.90	7.81	3.90	7.81	15.62	15.62

ND = Not determine.

In the checkerboard assay, a partial synergic interaction was observed in 3 combinations, including *P*. *akhurstii* subsp. *akhurstii* (bNN141.3_TH) plus oxacillin, *P*. *hainanensis* (bNN163.3_TH) plus oxacillin, and *P*. *akhurstii* subsp. *akhurstii* (bNN168.5_TH) plus oxacillin, with FIC index of 0.53, while the combination of one isolate of bacterial extract of *P*. *hainanensis* (bNN169.4_TH) plus oxacillin was shown as additive with FIC index at 1. Moreover, additive activity was seen in the combinations of 4 isolates of bacterial extracts (*P*. *akhurstii* subsp. *akhurstii* (bNN141.3_TH), *P*. *hainanensis* (bNN163.3_TH), *P*. *akhurstii* subsp. *akhurstii* (bNN168.5_TH) and *P*. *hainanensis* (bNN169.4_TH)) with vancomycin (FIC index = 1). Two independent experiments of checkerboard assay were the same result (Table 3).

**Table 3:** MIC and FIC indexes of Oxacillin and Vancomycin when used either alone or in combination with bacterial extracts against *S*. *aureus* strain PB36 (MRSA).

Combination of agents	MIC in combination (A+ B)	FIC index	Type of interaction
*P*. *akhurstii* subsp. *akhurstii* (bNN141.3_TH)	0.49	0.53	Partially synergistic
Oxacillin	0.0049		
*P*. *hainanensis* (bNN163.3_TH)	0.49	0.53	Partially synergistic
Oxacillin	0.0049		
*P*. *akhurstii* subsp. *akhurstii* (bNN168.5_TH)	0.49	0.53	Partially synergistic
Oxacillin	0.0049		
*P*. *hainanensis* (bNN169.4_TH)	0.153	1	Additive
Oxacillin	0.156		
*P*. *akhurstii* subsp. *akhurstii* (bNN141.3_TH)	0.0153	1	Additive
Vancomycin	0.003125		
*P*. *hainanensis* (bNN163.3_TH)	0.0153	1	Additive
Vancomycin	0.003125		
*P*. *akhurstii* subsp. *akhurstii* (bNN168.5_TH)	0.0153	1	Additive
Vancomycin	0.003125		
*P*. *hainanensis* (bNN169.4_TH)	0.0153	1	Additive
Vancomycin	0.003125		

FIC index = FIC A + FIC B, where FIC A is the MIC of bacterial extract in the combination/MIC of bacterial extract alone, and FIC B is the MIC of antibiotic in the combination/MIC of the antibiotic alone. The results were interpreted as follows: FICI ≤ 0.5, synergistic; 0.5 < FICI < 1, partially synergistic; FICI = 1, additive; >1 FICI ≤ 4, indifferent; and FICI > 4, antagonistic.

**Fig 2:**
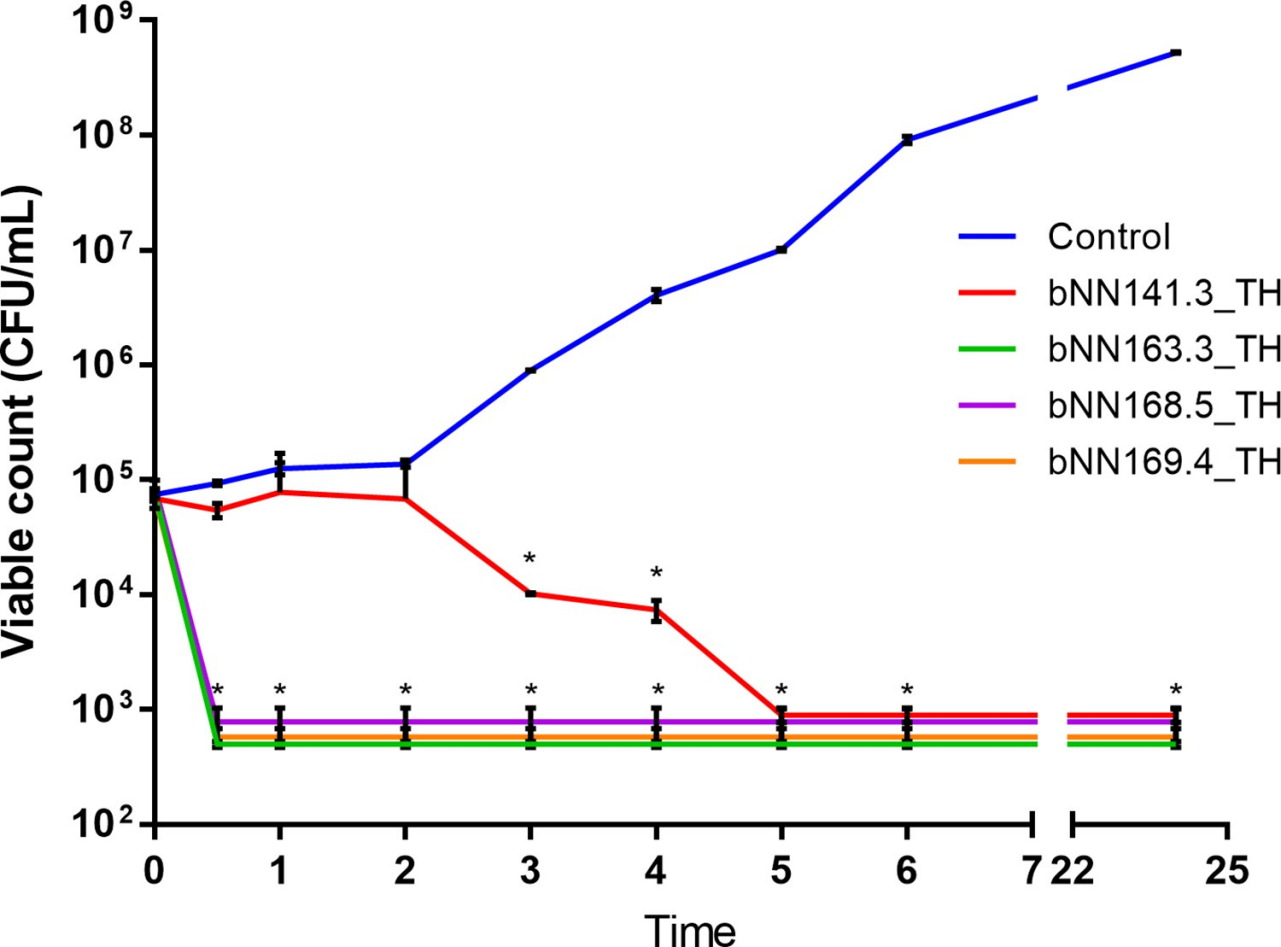
Time-kill curves for *S*. *aureus* strain PB36 (MRSA) using four extracts, including *P*. *akhurstii* subsp. *akhurstii* (bNN141.3_TH), *P*. *hainanensis* (bNN163.3_TH), *P*. *akhurstii* subsp. *akhurstii* (bNN168.5_TH) and *P*. *hainanensis* (bNN169.4_TH) compared with *S*. *aureus* strain PB36 (MRSA) cultured alone. Asterisk (*) indicates statistically significant differences between each *Photorhabdus* extracts and control (*p* < 0.001).

The results of the time-killing assay for bacterial extracts against *S*. *aureus* strain PB36 are shown in Fig 2. The extract of two isolates of *P*. *hainanensis* (bNN163.3_TH and bNN169.4_TH) and one isolate of *P*. *akhurstii* subsp. *akhurstii* (bNN168.5_TH) remarkably decreased cell viability from 10^5^ CFU/ml to 10^3^ CFU/ml by 30 min (P < 0.001, t-test), while the extract of *P*. *akhurstii* subsp. *akhurstii* (bNN141.3_TH), significantly reduced the viable bacteria after incubation for 3 h (P < 0.001, t-test) and gradually decreased cell viability to 10^3^ CFU/ml within 5 h. The untreated (controls) revealed no reduction in the viable count and steady growth throughout 24 h.

**Fig 3:**
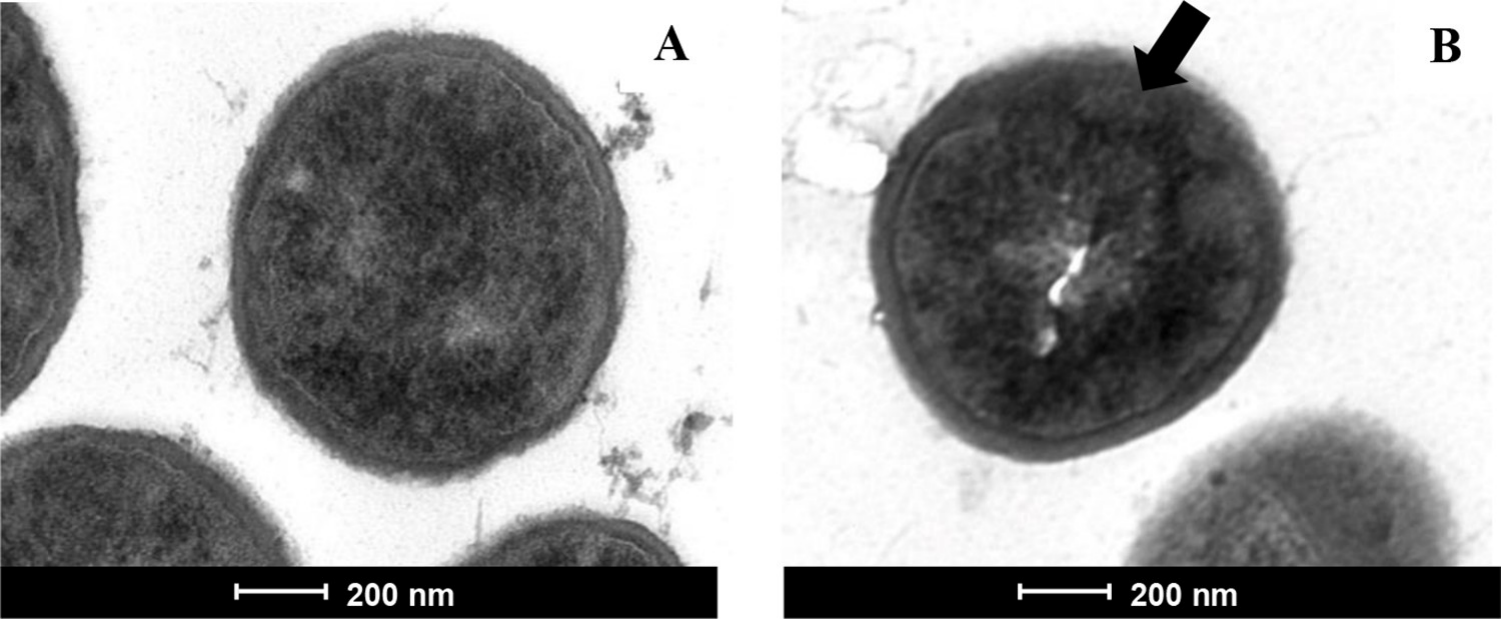
Transmission electron microscope for *S*. *aureus* strain PB36 (MRSA). Control (A) and treated with bacterial extract of *P*. *akhurstii* subsp. *akhurstii* (bNN168.5_TH) (B) (Magnification; A and B 20,000x, bar = 200 nm). Arrow indicates site of damage.

The antibacterial action of *P*. *akhurstii* subsp. *akhurstii* (bNN168.5_TH) extracts in inhibition the growth of *S*. *aureus* strain PB36 (MRSA) was primarily visualized by the transmission electron microscope (TEM). The TEM micrograph demonstrated that untreated control cell is intact morphology, and cell membrane and peptidoglycan were clearly defined. In treated cell, collapse, disruption, and 80% damage of cell membrane were observed. From the result suggested the mechanism of action of *P*. *akhurstii* subsp. *akhurstii* (bNN168.5_TH) was by inducing cell membrane damage (Fig 3).

**Fig 4:**
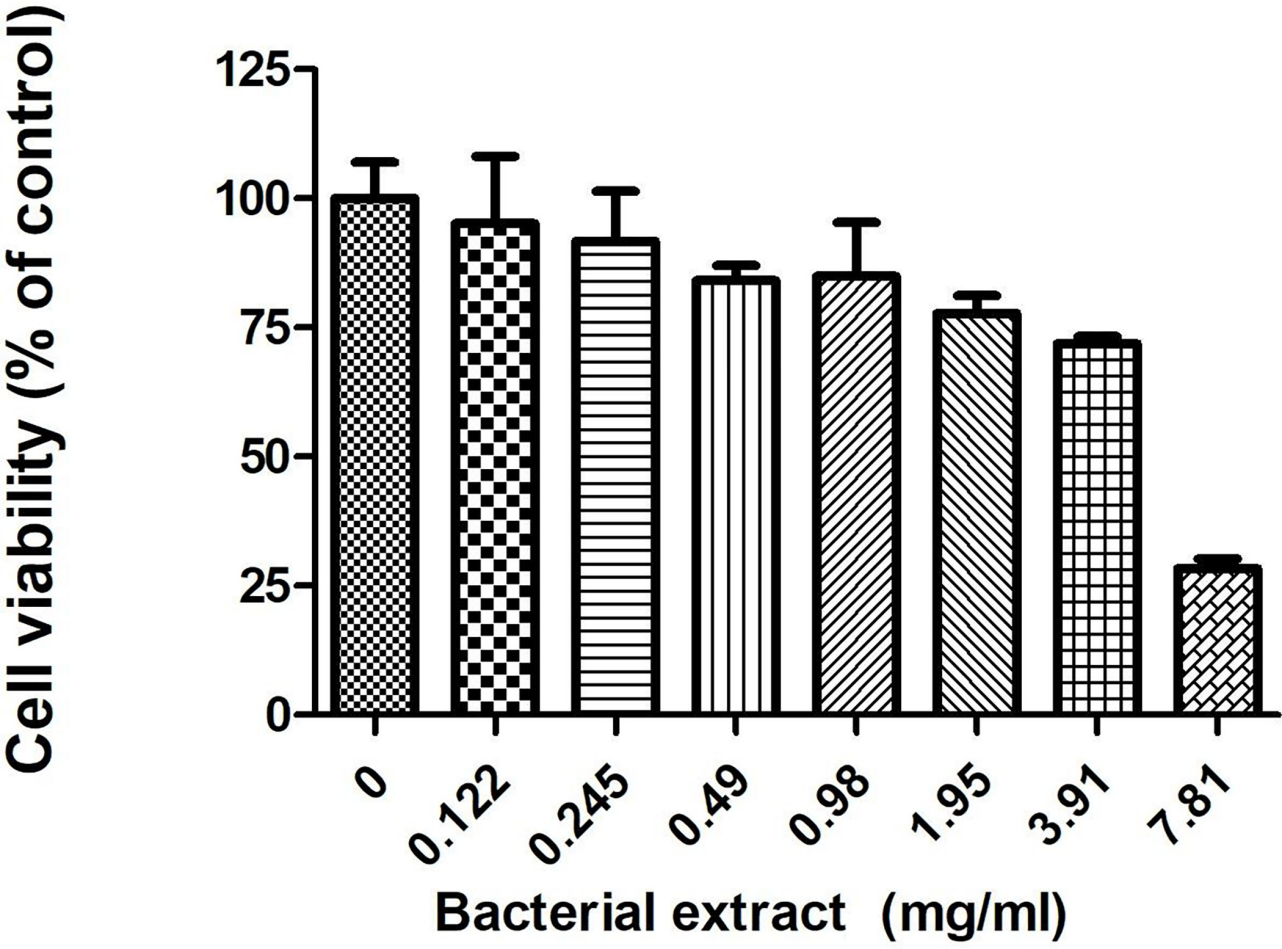
The effect of bacterial extract on the human hepatocellular carcinoma HepG2. Cells were treated with bacterial extract of *P*. *akhurstii* subsp. *akhurstii* (bNN168.5_TH) at concentrations in the range of 0 to 7.81 mg/ml.

The cytotoxic activity is shown in Fig 4. Bacterial extract of *P*. *akhurstii* subsp. *akhurstii* (bNN168.5_TH) at concentrations of 7.81 mg/ml exhibited cytotoxicity against the human liver cancer cell line (HepG2). On the other hand, 0.98 mg/ml of this bacterial extract showed that the effect of antibacterial activity had no cytotoxic effect. In our experiment, the concentration of this bacterial extract that caused the reduction of viable cells to 50% (IC50) was 4.35 mg/ml.

### Genome sequencing and annotation of *P*. *akhurstii* subsp. *akhurstii* (bNN168.5_TH)

**Fig 5:**
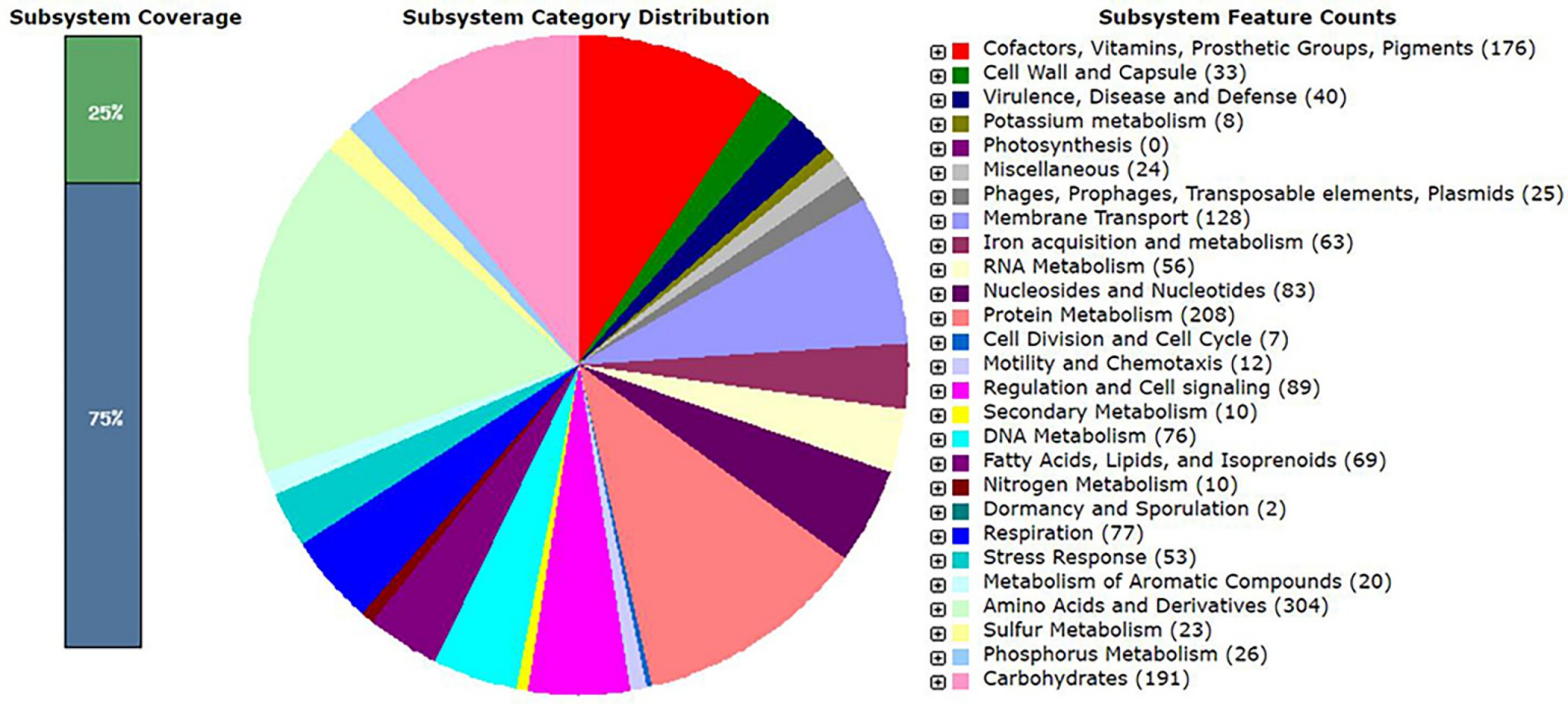
An overview of the subsystem category of the annotated whole-genome of *P*. *akhurstii* subsp. *akhurstii* (bNN168.5_TH) using RAST tool kit. The pie chart demonstrates the distribution of subsystem categories and the count of each subsystem feature. The bar graph demonstrates the subsystem coverage: 25% of coding sequences annotated in SEED subsystem features and 75% of coding sequences annotated outside of the SEED subsystem features.

*P*. *akhurstii* subsp. *akhurstii* (bNN168.5_TH) extract revealed the highest inhibitory activity against 5 strains of antibiotic-resistant bacteria (*S*. *aureus* strain PB36 (MRSA), *S*. *aureus* strain PB57 (MRSA), *A*. *baumannii* strain AB321 (MDR), *A*. *baumannii* strain AB322 (MDR) and *E*. *faecalis* ATCC51299). Therefore, the genome of this bacteria was selected to sequence. Of total 2,117,262,50 bases, 2,324,330 read counts with 151 bp read length were obtained as the raw sequence reads for the sequenced sample. The SPAdes genome assembler was employed for the *de novo* assembly after filtrating with 500 bp read length, which resulted in the generation of a total of 139 contigs with protein-encoding genes (PEGs) and 5.7 Mb size assembled data with a GC content of 42.70%. The estimated size of the genome was 5,695,571 bp as reported by the RAST tool kit results, and the contig L50 was found to be 11, whereas the N50 contig size was 16,1766 as presented in Table 4. Genome annotation was performed using the RAST tool kit, which resulted in the detection of 5,369 protein-coding sequences, 70 tRNA genes, and 10 rRNA operons. This draft genome was deposited at the NCBI-GenBank under the BioProject number PRJNA748897. The subsystems resulting from the RAST tool kit analysis are depicted in Fig 5.

**Table 4:** Genome information of *P*. *akhurstii* subsp. *akhurstii* (bNN168.5_TH).

Genome characteristic		length (bp)
**Estimated Genome size (bp)**		5,695,571
**Number of contig**		139
**Largest contig (bp)**		429,773
**N50 (bp)**		161,766
**N75 (bp)**		79,522
**L50**		11
**L75**		23
**G + C content (%)**		42.70
**Number of**		
	Coding sequence	5,369
	gene	4,906
	rRNA gene	10
	tRNA gene	70
	tmRNA	1

### Multiple genome comparison

**Fig 6:**
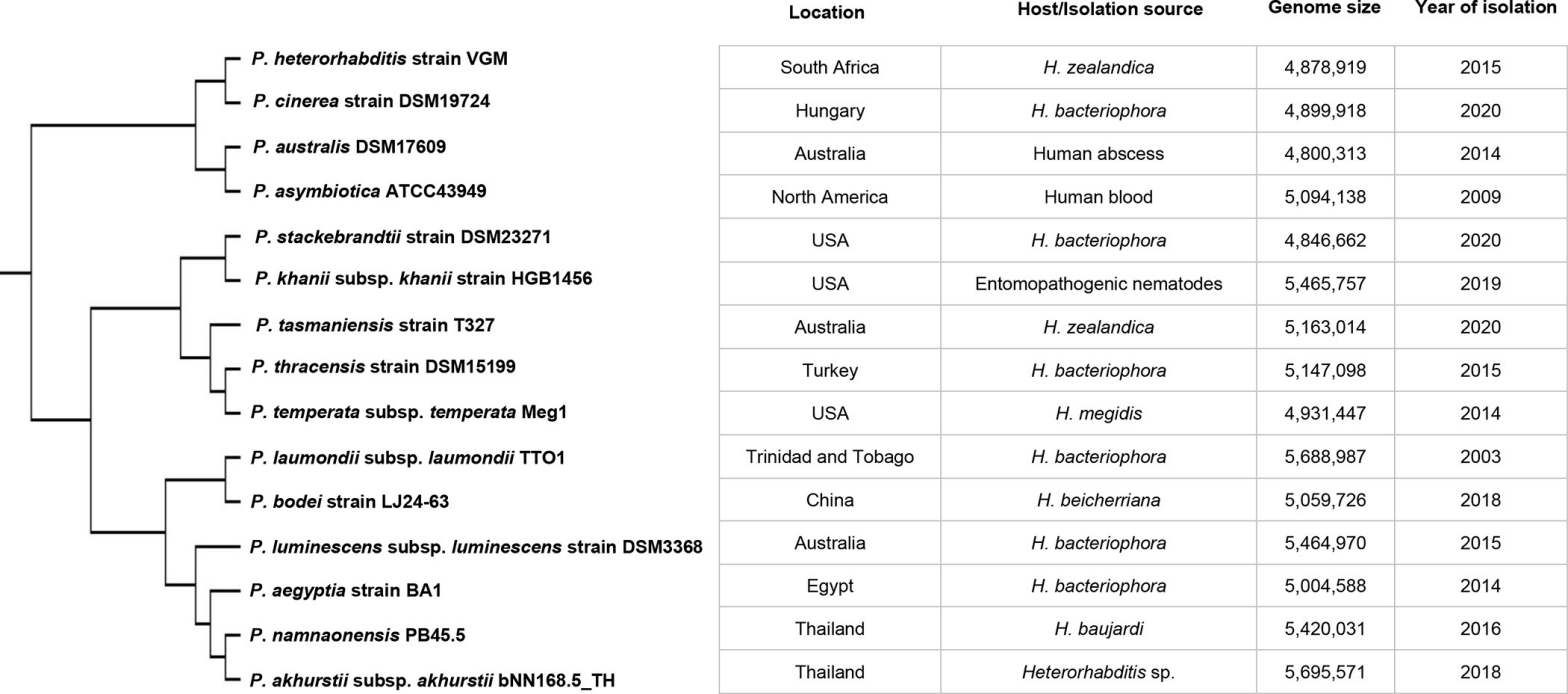
*In silico* analysis of the *P*. *akhurstii* subsp. *akhurstii* (bNN168.5_TH). Phylogenetic single-nucleotide polymorphism (SNP) tree of *P*. *akhurstii* subsp. *akhurstii* (bNN168.5_TH) with other *Photorhabdus* strains using Realphy.

For the determination of the evolutionary relationship of *P*. *akhurstii* subsp. *akhurstii* (bNN168.5_TH) with other *Photorhabdus* strains, whole-genome core SNP-based phylogenetic tree was constructed Phylogenetic analysis revealed that *P*. *akhurstii* subsp. *akhurstii* (bNN168.5_TH) was closely related to *P*. *namnaonensis* PB45.5. They were belonging to the cluster of *P*. *aegyptia* strain BA1, *P*. *luminescens* subsp. *luminescens* strain DSM3368, *P*. *bodei* strain LJ24-63 and *P*. *laumondii* subsp. *laumondii* TTO1 (Fig 6). Among genome sequence-published strains, *P*. *akhurstii* subsp. *akhurstii* (bNN168.5_TH) showed maximum average nucleotide identity (ANI) with *P*. *namnaonensis* PB45.5 (95.83%), *P*. *aegyptia* strain BA1 (95.67%), *P*. *bodei* strain LJ24-63 (92.36%), *P*. *laumondii* subsp. *laumondii* TTO1 (91.60%) and *P*. *luminescens* subsp. *luminescens* strain DSM3368 (91.59%).

### Identification of secondary metabolite‑biosynthetic gene clusters

Secondary metabolite‑biosynthetic gene clusters (BGCs) in the draft genome of *P*. *akhurstii* subsp. *akhurstii* (bNN168.5_TH) and other *Photorhabdus* strains were predicted using the AntiSMASH version 5.1.2.

**Fig 7:**
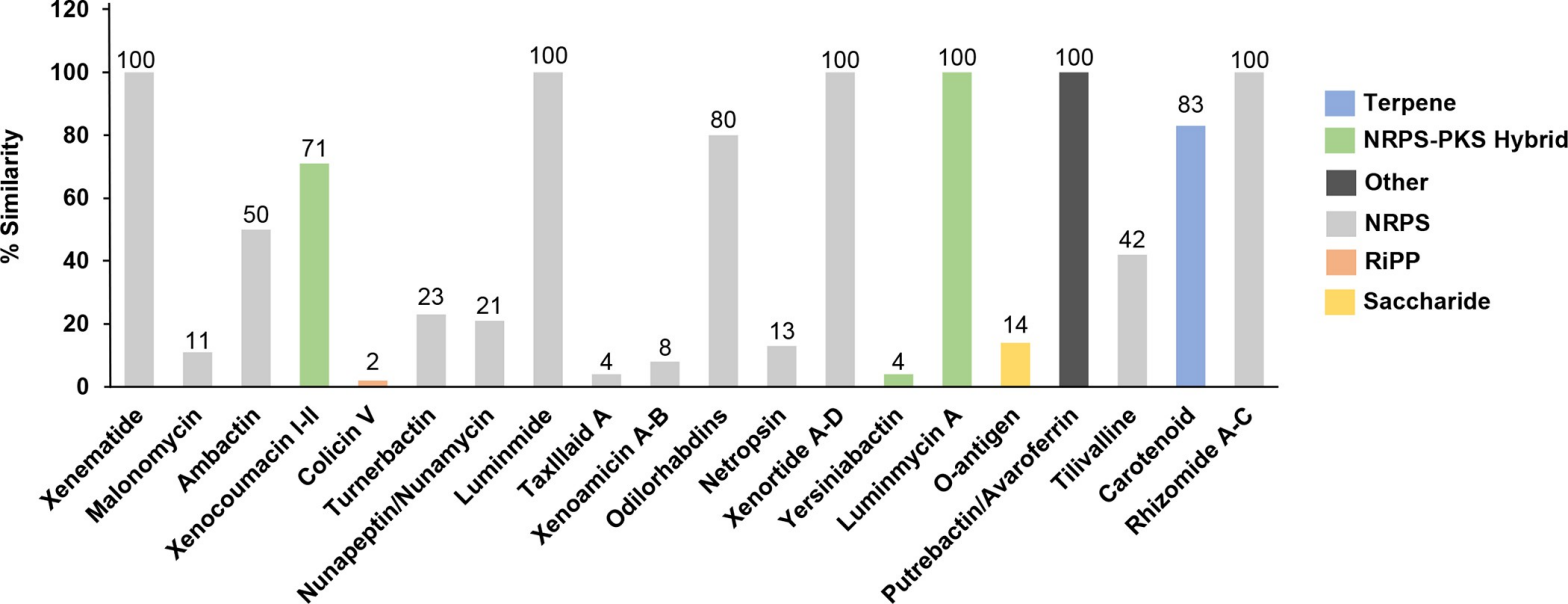
Secondary metabolite‑biosynthetic gene clusters (BGCs) in *P*. *akhurstii* subsp.*akhurstii* (bNN168.5_TH) draft genome. Bar color indicates that classification of each cluster type and the number above the bars indicate percent similarity.

**Fig 8:**
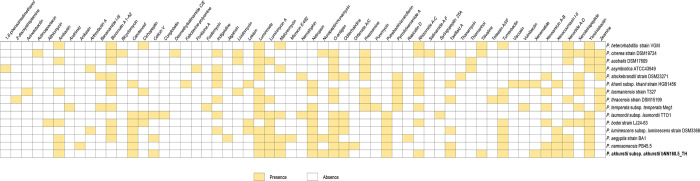
Secondary metabolite biosynthesis gene clusters of *P*. *akhurstii* subsp. *akhurstii* and other *Photorhabdus* strains were identified using antiSMASH (Version 5.1.2) and R studio version 1.4.1717 with heatmap and tidyverse packages.

The results revealed that *P*. *akhurstii* subsp. *akhurstii* (bNN168.5_TH) can synthesize abundant secondary metabolites, which might be an important source of novel bioactive compounds. The completely sequenced biosynthetic gene clusters were predicted in *P*. *akhurstii* subsp. *akhurstii* (bNN168.5_TH) genome contained with non-ribosomal peptide synthetase cluster (NRPS), hybrid NRPS-type I polyketide synthase (PKS), terpene, saccharide, ribosomally synthesized post-translationally modified peptide product (RiPP) and other. The biosynthetic genes were observed in *P*. *akhurstii* subsp. *akhurstii* (bNN168.5_TH) genome with 100% similarity, which known bioactive compounds as xenematide, luminmide, xenortide A-D, luminmycin A, putrebactin/avaroferrin and rhizomide A-C. In addition, the other types of known BGCs (carotenoid, xenocoumacin I-II, ambactin, tilivalline, turnerbactin, nunapeptin/ nunamycin, O-antigen, netropsin, malonomycin, xenoamicin A-B, taxlllaid A, yersiniabactin, and colicin V) were observed in the genome with 2–83% similarity (Fig 7, and S1 Table). The details of the location of all sequenced biosynthetic gene clusters showed in S1 Fig. Furthermore, BGCs identified in *P*. *akhurstii* subsp. *akhurstii* (bNN168.5_TH) were predicted as the closely related *Photorhabdus* strains, except Xenematide and Tilivalline (Fig 8).

**Table 5** showed the distribution of biosynthetic gene clusters (BGCs) in 15 genomes of *Photorhabdus*. The non-ribosomal peptide synthetase cluster (NRPS) is one of the most abundant BGCs present in the *Photorhabdus* strains, which includes 145 cluster. *P*. *akhurstii* subsp. *akhurstii* (bNN168.5_TH) and *P*. *luminescens* subsp. *luminescens* strain DSM3368 were detected 13 clusters of NRPS higher than *P*. *australis* DSM17609 (13), which was detected 5 clusters. About 23 hybrid BGCs were detected from the 13 *Photorhabdus* genomes. These hybrid clusters were formed by the combination of two different types of BGCs and could be as simple as commonly observed T1PKS-NRPS hybrids. Besides, the other hybrid clusters either involved a PKS or an NRPS cluster in combination with other types (NRP:Beta-lactam + Polyketide:Type II, NRP + Polyketide:Modular type I + Polyketide:PUFA synthase or related etc.). It is detected from 7 *Photorhabdus* genomes. Moreover, 4 BGCs such as siderophore, resorcinol, terpene and thiopeptide were detected in *P*. *akhurstii* subsp. *akhurstii* bNN168.5_TH (1), *P*. *namnaonensis* PB45.5 (2), *P*. *aegyptia* strain BA1 (3), *P*. *luminescens* subsp. *luminescens* strain DSM3368 (4) and *P*. *bodei* strain LJ24-63 (5). For the other cluster type (Phosphonate, Nucleoside and tRNA-derived) was found in 3 *Photorhabdus* genomes including *P*. *temperata* subsp. *temperata* Meg1 (7), *P*. *khanii* subsp. *khanii* strain HGB1456 (10) and *P*. *cinerea* strain DSM19724 (14).

**Table 5:** The distribution of biosynthetic gene clusters (BGCs) in 15 genomes of *Photorhabdus*. Species: *P*. *akhurstii* subsp. *akhurstii* bNN168.5_TH (1), *P*. *namnaonensis* PB45.5 (2), *P*. *aegyptia* strain BA1 (3), *P*. *luminescens* subsp. *luminescens* strain DSM3368 (4), *P*. *bodei* strain LJ24-63 (5), *P*. *laumondii* subsp. *laumondii* TTO1 (6), *P*. *temperata* subsp. *temperata* Meg1 (7), *P*. *thracensis* strain DSM15199 (8), *P*. *tasmaniensis* strain T327 (9), *P*. *khanii* subsp. *khanii* strain HGB1456 (10), *P*. *stackebrandtii* strain DSM23271 (11), *P*. *asymbiotica* ATCC43949 (12), *P*. *australis* DSM17609 (13), *P*. *cinerea* strain DSM19724 (14) and *P*. *heterorhabditis* strain VGM (15).

Cluster Type/Species	1	2	3	4	5	6	7	8	9	10	11	12	13	14	15	Total
NRPS	13	9	10	13	10	11	10	8	10	11	11	6	5	10	8	145
T1PKS_NRPS	3	4	1	3	2	1	0	1	1	1	0	2	2	1	1	23
T1PKS	0	0	0	0	0	0	0	0	0	0	1	0	0	0	0	1
Siderophore	1	1	1	1	1	1	0	0	0	0	0	0	0	0	0	6
Resorcinol	1	1	1	1	1	0	0	0	1	0	0	0	1	0	1	8
Terpene	1	1	1	1	1	1	0	0	0	1	0	0	0	0	0	7
Bacteriocin	0	0	1	0	0	0	0	0	0	0	0	0	0	1	0	2
Thiopeptide	1	1	1	1	1	1	0	1	1	0	1	1	1	0	0	11
Amglyccycl	0	0	0	0	0	0	0	1	1	0	0	0	1	2	0	5
Other hybrids	0	0	0	0	0	0	2	1	1	0	1	1	1	1	0	8
Other	0	0	0	0	0	0	2	0	0	1	0	0	0	1	0	4
**Total**	20	17	18	20	16	16	14	12	15	14	14	10	11	16	10	220

## Discussion

*Xenorhabdus* and *Photorhabus* have been reported to produce antibacterial compounds. In the present study, four extracts of *Photorhabdus* isolates showed high antibacterial potential against many antibiotic-resistant bacteria. The *Photorhabdus akhurstii* subsp. *akhurstii* (bNN168.5_TH) extract had the most inhibitory effect compared to the bacterial isolates tested. The previous study showed that *P*. *luminescens* could inhibit the growth of *B*. *subtilis*, *E*. *coli*, *S*. *pyogenes* and *S*. *aureus* RN4220 (drug-resistant and clinical isolate) [35]. *Trans*-cinnamic acid (TCA), produced by *Photorhabdus* could inhibit the growth of *Colletotrichum gloeosporioides*, *C*. *fragariae*, and *C*. *acutatum* and *Fusicladium effusum*, which is the cause of Pecan scab [29]. In addition, our previous study demonstrated that the *P*. *temperata* subsp. *temperata* (bMW27.4_TH) extract could inhibit up to 10 strains of antibiotic-resistant bacteria. All bacterial extracts from *Photorhabdus* of the Mae Wong National Park, and *P*. *luminescens* of Saraburi province could inhibit the growth of many strains of antibiotic-resistant bacteria, including *S*. *aureus* ATCC20475, *S*. *aureus* strain PB36 (MRSA), and *S*. *aureus* strain PB57 (MRSA), *A*. *baumannii* strain AB320 (XDR), *A*. *baumannii* strain AB321 (MDR), *A*. *baumannii* strain AB322 (XDR), *E*. *faecalis* ATCC51299, and *K*. *pneumoniae* strain PB21 (ESBL and CRE) [30,31]. *Xenorhabdus*-produced xenocoumacin [43] and amicoumacin derivatives [44] were found to be potent antibiotics against *S*. *aureus* [18], while all the *Photorhabdus* spp. produced isopropylstilbene [30,45,46] which has various biological activities, including antibiotic activity against *S*. *aureus* and *E*. *coli* [47].

Based on the MIC and MBC, the ability to inhibit the growth of antibiotic-resistant bacteria varied with different isolates of *Photorhabdus*. This may be due to either the ability of each symbiotic bacterium to produce effective metabolites or the susceptibility of antibiotic-resistant bacteria. The MIC and MBC of *P*. *luminescens* extracts on *S*. *aureus* strain PB36 (MRSA) was found in 0.98 mg/ml in this study. Similar to our previous study demonstrated that the MIC and MBC of *P*. *luminescens* extracts on *S*. *aureus* strain PB36 had 0.98 mg/ml [31]. In contrast, the *Stephania suberosa* Forman extract (SSE) against ampicillin-resistant *S*. *aureus* had a higher MIC with 4 mg/ml [48]. High MIC was also noted in the olive oil polyphenol extract [49], *Camellia sinensis* and *Azadirachta indica* leaves extracts [3] against *S*. *aureus*. This indicates that *Photorhabdus* extracts are more effective than those of SSE, olive oil, polyphenol extract, *Camellia sinensis* and *Azadirachta indica* leaves extracts.

The combination of bacterial extracts and antibiotics (oxacillin and vancomycin) exhibited partially synergistic and additive activity against the *S*. *aureus* strain PB36 (MRSA). These results are in contrast with the previous studies of Teethaisong et al. [40], who reported that the combination of *Boesenbergia rotunda* (L.) Mansf. extract and vancomycin exhibited no synergistic activity against all staphylococci tested, including *S*. *aureus* ATCC29213.

In terms of the time-kill assay for *S*. *aureus* strain PB36 (MRSA), the extracts of *P*. *akhurstii s* subsp. *akhurstii* (bNN141.3_TH), *P*. *hainanensis* (bNN163.3_TH), *P*. *akhurstii* subsp. *akhurstii* (bNN168.5_TH) and *P*. *hainanensis* (bNN169.4_TH) have stronger bactericidal activities. This result was correlated to the MIC and MBC assays for *S*. *aureus* isolates investigated. The number of *S*. *aureus* strain PB36 (MRSA) was rapidly reduced after exposure to the extracts of *P*. *hainanensis* (bNN163.3_TH), *P*. *akhurstii* subsp. *akhurstii* (bNN168.5_TH), and *P*. *hainanensis* (bNN169.4_TH), while number of *S*. *aureus* strain PB36 (MRSA) was reduced within 5 h after exposure to the extract of *P*. *akhurstii* subsp. *akhurstii* (bNN141.3_TH). Similar to our previous report showed that the number of *S*. *aureus* PB36 (MRSA) was reduced within 30 min after exposure to the extract of *P*. *akhurstii s* subsp. *akhurstii* [31]. However, it differs from the previous findings, wherein the *Stephania suberosa* Forman extract plus ampicillin antibiotic exhibited synergistic activity against the ampicillin-resistant *S*. *aureus* [48]. Apart from this, the combinations of *Cyperus rotundus* L. extract and ampicillin antibiotics showed that the killing of ampicillin-resistant *S*. *aureus* cells was dramatically reduced by these combinations [50].

A transmission electron microscope, after treating the cell of *S*. *aureus* strain PB36 with *P*. *akhurstii* subsp. *akhurstii* (bNN168.5_TH) extract), found the cell membrane damage when compared with control. These results are consistent with those of [40,48] and Cheypratub et al. [50] that *Stephania suberosa* Forman extract plus ampicillin, *Boesenbergia rotunda* (L.) Mansf. extract plus cloxacillin and *Cyperus rotundus* L. extract plus ampicillin inhibited of *S*. *aureus*. The target of *P*. *akhurstii* subsp. *akhurstii* (bNN168.5_TH) extract was bacterial membrane, while oxacillin is well-known targeting peptidoglycan. This finding could explain the partial synergistic interaction by inhibiting the growth of bacteria at different sites of action [40]_._

The MIC (0.98 mg/ml) of bacterial extract of *P*. *akhurstii* subsp. *akhurstii* (bNN168.5_TH) against antibiotic-resistant bacteria did not affect the viability of HepG2 cell line. These desired properties of antibacterial compounds are the selective inhibition against bacteria with less cytotoxic effect on normal cells for avoiding side effects to healthy tissues [51,52].

In this work, we report the first draft genome sequence and identify the biosynthetic gene clusters (BGCs) in the *P*. *akhurstii* subsp. *akhurstii* from Thai strain. A detailed analysis of the genome of *P*. *akhurstii* subsp. *akhurstii* (bNN168.5_TH) predicted non-ribosomal peptide synthetase cluster (NRPS), hybrid NRPS-type I polyketide synthase (PKS) and siderophore, which was consistent with Bozhuyuk et al. [53], who reported that the main BGCs were NRPs detected on *Photorhabdus*. In addition, the genome sequence of *Photorhabdus* and *Xenorhabdus* was very similar. Several BGCs (xenematide, ambactin, xenocoumacin, xenoamicin, xenortide, and tilivalline) of *Xenorhabdus* were found in *Photorhabdus* strains [37].

In summary, *Photorhabdus* spp. showed the potential to inhibit the growth of *S*. *aureus* strain PB36 (MRSA). This may be at least one of the major mechanisms of action of the *Photorhabdus* extract against antibiotic-resistant bacteria and is useful in further drug discovery from natural resources.

## Supporting information

S1 Fig The details of the location of all sequenced biosynthetic gene clusters. (DOCX)

S1 Table The location of gene clusters; non-ribosomal peptide synthetase cluster (NRPS), hybrid NRPS-type l polyketide synthase (PKS) and siderophore of *P*. *akhurstii* subsp. *akhurstii* (bNN168.5_TH) and the similarity percentage with known clusters. (DOCX)
