# Supplementary material for: Genome analysis of secondary metabolite‑biosynthetic gene clusters of Photorhabdus akhurstii subsp. akhurstii and its antibacterial activity against antibiotic-resistant bacteria
Source: PLoS One. 2022 Sep 21;17(9):e0274956. doi: 10.1371/journal.pone.0274956 (PMC9491552; doi:10.1371/journal.pone.0274956)
Supplement: S1 Fig — (DOCX) [file pone.0274956.s001.docx]

**
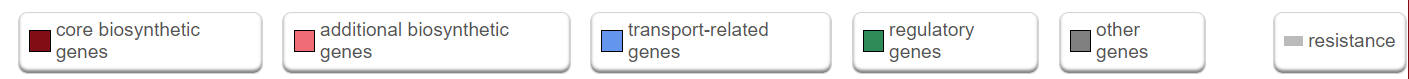
**

**
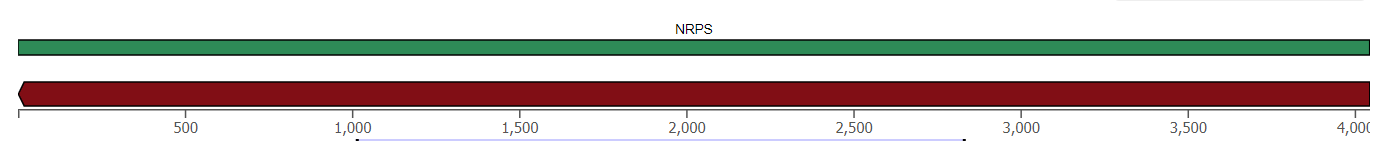
Region 3.1**

**
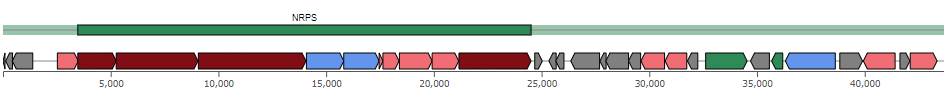
Region 5.1**

**
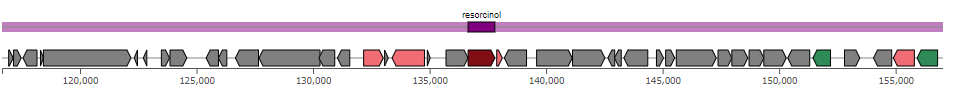
Region 30.2**

**Region 35.1**


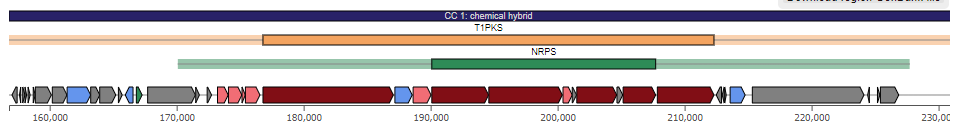


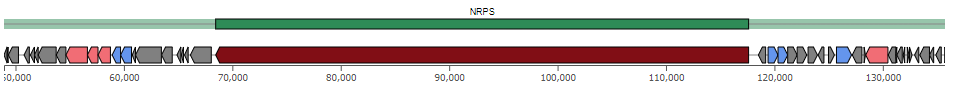
**Region 36.1**

**
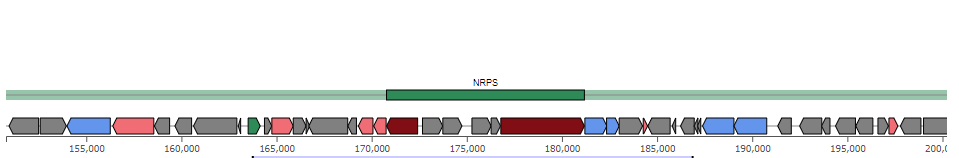
Region 36.2**

**
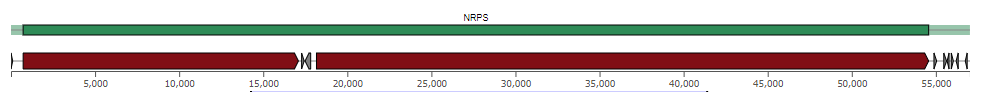
Region 39.1**

**
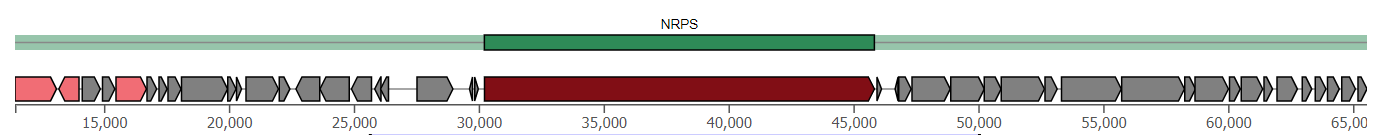
Region 42.1**

**
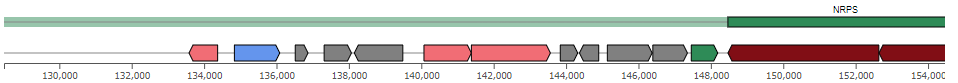
Region 42.2**

**
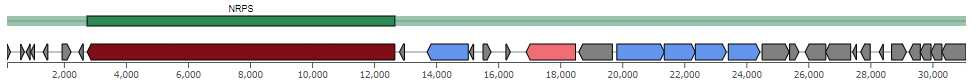
Region 46.1**

**
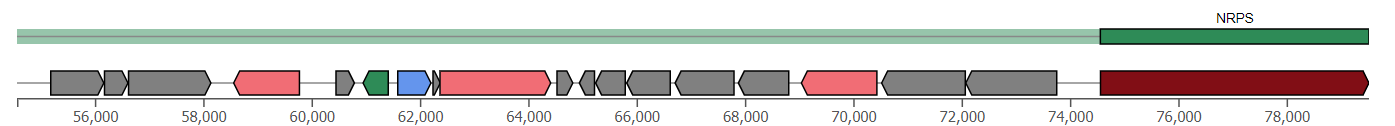
Region 46.2**

**
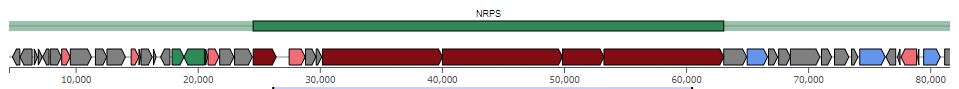
Region 51.1**

**
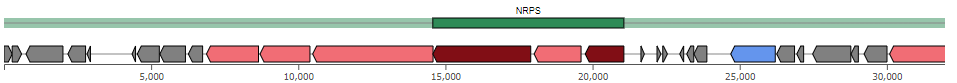
Region 52.1**

**
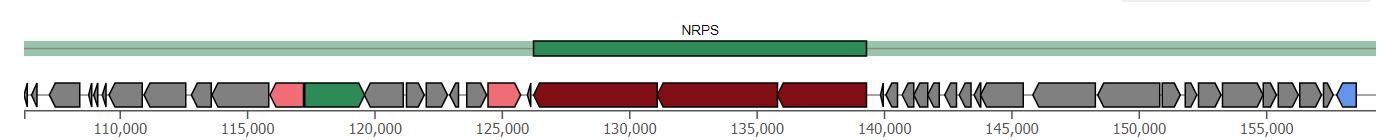
Region 54.1**

**
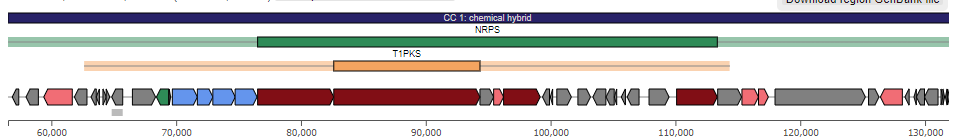
Region 73.1**

**
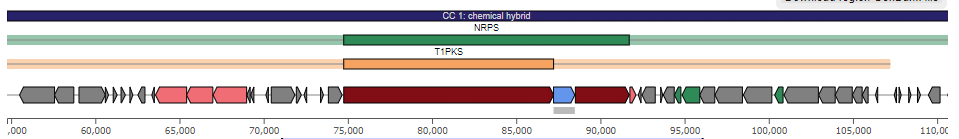
Region 74.1**

**
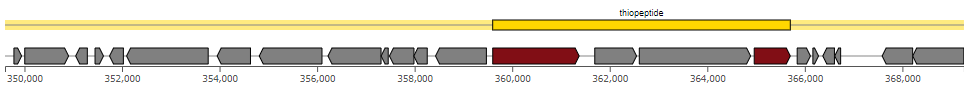
Region 74.2**

**
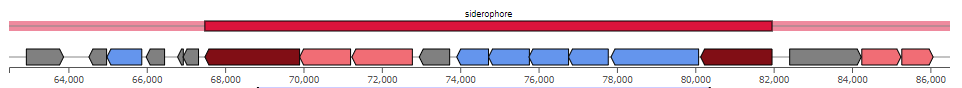
Region 81.1**

**
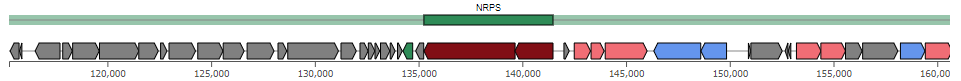
Region 81.2**

**
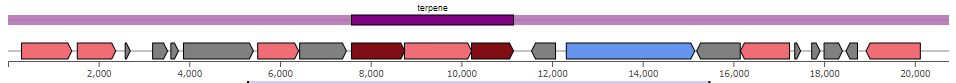
Region 89.1**

**Region 95.1**


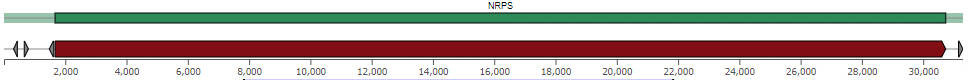


**S1 Fig.** The details of the location of all sequenced biosynthetic gene clusters.
