# Supplementary material for: Genome analysis of secondary metabolite‑biosynthetic gene clusters of Photorhabdus akhurstii subsp. akhurstii and its antibacterial activity against antibiotic-resistant bacteria
Source: PLoS One. 2022 Sep 21;17(9):e0274956. doi: 10.1371/journal.pone.0274956 (PMC9491552; doi:10.1371/journal.pone.0274956)
Supplement: S1 Table — (DOCX) [file pone.0274956.s002.docx]

| **Region** | **Type** | **From** | **To** | **Most similar known cluster** | | **Similarity** |
| --- | --- | --- | --- | --- | --- | --- |
| [3.1](https://usegalaxy.eu/datasets/11ac94870d0bb33a482fbbf0c81808f9/display/?preview=True#r3c1) | [NRPS](https://docs.antismash.secondarymetabolites.org/glossary/#nrps) | 1 | 4,046 | [xenematide](https://mibig.secondarymetabolites.org/go/BGC0001825/1) | NRP | 100% |
| [5.1](https://usegalaxy.eu/datasets/11ac94870d0bb33a482fbbf0c81808f9/display/?preview=True#r5c1) | [NRPS](https://docs.antismash.secondarymetabolites.org/glossary/#nrps) | 1 | 44,512 | [malonomycin](https://mibig.secondarymetabolites.org/go/BGC0001942/1) | NRP + Polyketide | 11% |
| [30.2](https://usegalaxy.eu/datasets/11ac94870d0bb33a482fbbf0c81808f9/display/?preview=True#r30c2) | [resorcinol](https://docs.antismash.secondarymetabolites.org/glossary/#resorcinol) | 116,653 | 157,801 | [ambactin](https://mibig.secondarymetabolites.org/go/BGC0001131/1) | NRP | 50% |
| [35.1](https://usegalaxy.eu/datasets/11ac94870d0bb33a482fbbf0c81808f9/display/?preview=True#r35c1) | [T1PKS](https://docs.antismash.secondarymetabolites.org/glossary/#t1pks),[NRPS](https://docs.antismash.secondarymetabolites.org/glossary/#nrps) | 156,797 | 232,312 | [xenocoumacin 1 / xenocoumacin II](https://mibig.secondarymetabolites.org/go/BGC0001054/1) | NRP + Polyketide:Modular type I | 71% |
| [36.1](https://usegalaxy.eu/datasets/11ac94870d0bb33a482fbbf0c81808f9/display/?preview=True#r36c1) | [NRPS](https://docs.antismash.secondarymetabolites.org/glossary/#nrps) | 48,940 | 137,390 | [colicin V](https://mibig.secondarymetabolites.org/go/BGC0001555/1) | RiPP | 2% |
| [36.2](https://usegalaxy.eu/datasets/11ac94870d0bb33a482fbbf0c81808f9/display/?preview=True#r36c2) | [NRPS](https://docs.antismash.secondarymetabolites.org/glossary/#nrps) | 150,782 | 201,180 | [turnerbactin](https://mibig.secondarymetabolites.org/go/BGC0000451/1) | NRP | 23% |
| [39.1](https://usegalaxy.eu/datasets/11ac94870d0bb33a482fbbf0c81808f9/display/?preview=True#r39c1) | [NRPS](https://docs.antismash.secondarymetabolites.org/glossary/#nrps) | 1 | 57,026 | [nunapeptin / nunamycin](https://mibig.secondarymetabolites.org/go/BGC0001416/1) | NRP | 21% |
| [42.1](https://usegalaxy.eu/datasets/11ac94870d0bb33a482fbbf0c81808f9/display/?preview=True#r42c1) | [NRPS](https://docs.antismash.secondarymetabolites.org/glossary/#nrps) | 11,440 | 65,562 | [luminmide](https://mibig.secondarymetabolites.org/go/BGC0001128/1) | NRP | 100% |
| [42.2](https://usegalaxy.eu/datasets/11ac94870d0bb33a482fbbf0c81808f9/display/?preview=True#r42c2) | [NRPS](https://docs.antismash.secondarymetabolites.org/glossary/#nrps) | 128,472 | 154,964 | [taxlllaid A](https://mibig.secondarymetabolites.org/go/BGC0001133/1) | NRP | 4% |
| [46.1](https://usegalaxy.eu/datasets/11ac94870d0bb33a482fbbf0c81808f9/display/?preview=True#r46c1) | [NRPS](https://docs.antismash.secondarymetabolites.org/glossary/#nrps) | 166 | 31,081 | [xenoamicin A / xenoamicin B](https://mibig.secondarymetabolites.org/go/BGC0000464/1) | NRP:Cyclic depsipeptide | 8% |
| [46.2](https://usegalaxy.eu/datasets/11ac94870d0bb33a482fbbf0c81808f9/display/?preview=True#r46c2) | [NRPS](https://docs.antismash.secondarymetabolites.org/glossary/#nrps) | 54,563 | 79,522 | [xenematide](https://mibig.secondarymetabolites.org/go/BGC0001825/1) | NRP | 100% |
| [51.1](https://usegalaxy.eu/datasets/11ac94870d0bb33a482fbbf0c81808f9/display/?preview=True#r51c1) | [NRPS](https://docs.antismash.secondarymetabolites.org/glossary/#nrps) | 4,542 | 83,090 | [odilorhabdins](https://mibig.secondarymetabolites.org/go/BGC0001716/1) | NRP | 80% |
| [52.1](https://usegalaxy.eu/datasets/11ac94870d0bb33a482fbbf0c81808f9/display/?preview=True#r52c1) | [NRPS](https://docs.antismash.secondarymetabolites.org/glossary/#nrps) | 1 | 32,585 | [netropsin](https://mibig.secondarymetabolites.org/go/BGC0000327/1) | NRP | 13% |
| [54.1](https://usegalaxy.eu/datasets/11ac94870d0bb33a482fbbf0c81808f9/display/?preview=True#r54c1) | [NRPS](https://docs.antismash.secondarymetabolites.org/glossary/#nrps) | 106,253 | 159,321 | [xenortide A / xenortide B / xenortide C / xenortide D](https://mibig.secondarymetabolites.org/go/BGC0000465/1) | NRP | 100% |

**S1 Table.** The location of gene clusters; non-ribosomal peptide synthetase cluster (NRPS), hybrid NRPS-type l polyketide synthase (PKS) and siderophore of *P. akhurstii* subsp. *akhurstii* (bNN168.5_TH) and the similarity percentage with known clusters.

**S1 Table. (cont.)**

| **Region** | **Type** | **From** | **To** | **Most similar known cluster** | | **Similarity** |
| --- | --- | --- | --- | --- | --- | --- |
| [73.1](https://usegalaxy.eu/datasets/11ac94870d0bb33a482fbbf0c81808f9/display/?preview=True#r73c1) | [NRPS](https://docs.antismash.secondarymetabolites.org/glossary/#nrps),[T1PKS](https://docs.antismash.secondarymetabolites.org/glossary/#t1pks) | 56,528 | 133,357 | [yersiniabactin](https://mibig.secondarymetabolites.org/go/BGC0001055/1) | NRP + Polyketide | 4% |
| [74.1](https://usegalaxy.eu/datasets/11ac94870d0bb33a482fbbf0c81808f9/display/?preview=True#r74c1) | [NRPS](https://docs.antismash.secondarymetabolites.org/glossary/#nrps),[T1PKS](https://docs.antismash.secondarymetabolites.org/glossary/#t1pks) | 54,756 | 111,711 | [luminmycin A](https://mibig.secondarymetabolites.org/go/BGC0000383/1) | NRP + Polyketide:Modular type I | 100% |
| [74.2](https://usegalaxy.eu/datasets/11ac94870d0bb33a482fbbf0c81808f9/display/?preview=True#r74c2) | [thiopeptide](https://docs.antismash.secondarymetabolites.org/glossary/#thiopeptide) | 349,601 | 369,262 | [O-antigen](https://mibig.secondarymetabolites.org/go/BGC0000781/1) | Saccharide | 14% |
| [81.1](https://usegalaxy.eu/datasets/11ac94870d0bb33a482fbbf0c81808f9/display/?preview=True#r81c1) | [siderophore](https://docs.antismash.secondarymetabolites.org/glossary/#siderophore) | 62,484 | 86,959 | [putrebactin / avaroferrin](https://mibig.secondarymetabolites.org/go/BGC0001870/1) | Other | 100% |
| [81.2](https://usegalaxy.eu/datasets/11ac94870d0bb33a482fbbf0c81808f9/display/?preview=True#r81c2) | [NRPS](https://docs.antismash.secondarymetabolites.org/glossary/#nrps) | 115,258 | 161,480 | [tilivalline / 9-deoxy tilivalline / dihydroxy tilivalline / dehydro tilivalline / dihydroxy-dehydro tilivalline](https://mibig.secondarymetabolites.org/go/BGC0000446/1) | NRP:Pyrrolobenzodiazepine | 42% |
| [89.1](https://usegalaxy.eu/datasets/11ac94870d0bb33a482fbbf0c81808f9/display/?preview=True#r89c1) | [terpene](https://docs.antismash.secondarymetabolites.org/glossary/#terpene) | 1 | 21,150 | [carotenoid](https://mibig.secondarymetabolites.org/go/BGC0000640/1) | Terpene | 83% |
| [95.1](https://usegalaxy.eu/datasets/11ac94870d0bb33a482fbbf0c81808f9/display/?preview=True#r95c1) | [NRPS](https://docs.antismash.secondarymetabolites.org/glossary/#nrps) | 1 | 31,297 | [rhizomide A / rhizomide B / rhizomide C](https://mibig.secondarymetabolites.org/go/BGC0001758/1) | NRP | 100% |
